# Assessing Weight Stigma Interventions: A Systematic Review of Randomized Controlled Trials

**DOI:** 10.1007/s13679-025-00628-w

**Published:** 2025-04-14

**Authors:** Christy Wang, William D. Murley, Sameeksha Panda, Caroline A. Stiver, Cambria L. Garell, Tannaz Moin, Amanda K. Crandall, A. Janet Tomiyama

**Affiliations:** 1 Department of Psychology, University of California, Los Angeles, CA 90095 USA; 2 Department of Pediatrics, David Geffen School of Medicine, University of California, Los Angeles, CA 90095 USA; 3 Divisions of Endocrinology, Diabetes & Metabolism and General Internal Medicine & Health Services Research, David Geffen School of Medicine, University of California, Los Angeles, CA 90095 USA; 4 VA Health Services Research and Development (HSR&D) Center for Healthcare Innovation, Implementation, and Policy, VA Greater los Angeles Healthcare System, Los Angeles, CA 90073 USA; 5 Department of Pediatrics, Division of Developmental Behavioral Pediatrics, University of Michigan Medical School, Ann Arbor, MI 48104 USA

**Keywords:** Weight Stigma, Weight Bias, Weight Discrimination, Prevention, Intervention, Systematic Review

## Abstract

**Purpose of Review:**

The primary goals of this pre-registered systematic review were to critically evaluate the existing randomized controlled trials targeting weight stigma/bias and identify promising avenues for future research.

**Recent Findings:**

Prior systematic reviews have highlighted intervention strategies such as shifting causal attributions of obesity, evoking empathy, deploying weight-inclusive approaches, increasing education, and combining these strategies. Here, we provide an updated systematic review of weight stigma interventions.

**Summary:**

A systematic search was conducted following the PRISMA guidelines and performed in PubMed/Medline, PubMed, PsycINFO, and Google Scholar until October 2024, yielding a final sample of 56 articles. In addition to previously established strategies, we identified several novel strategies, such as cognitive dissonance and connection building. Interventions can largely shift attitudinal outcomes, but future research should extend beyond attitude measures, assess weight bias over a longer term, and across more diverse populations.

**Supplementary Information:**

The online version contains supplementary material available at 10.1007/s13679-025-00628-w.

## Introduction

Reported instances of weight stigma have risen from an estimated 12% in 2004–2006 to 40% in 2021 [1, 2]. Weight stigma can be broadly defined as the social devaluation of individuals based on their body size or weight [3]. It has consequences across virtually every life domain [4], including healthcare [5], education [6], relationships [7], and employment [2]. Studies find nearly all individuals of higher weight experience weight stigma during their lifetime [5, 7]. Moreover, weight stigma does not appear to discriminate by age, gender, or even body weight [2, 8].

Weight stigma is associated with many negative health outcomes. It is correlated with an increased risk of obesity, cardiovascular disease, and diabetes [9], as well as increases in mortality, even after accounting for factors like body mass index (BMI) [10]. Weight stigma contributes to increased anxiety, depression, body dissatisfaction, low self-esteem, and a heightened risk of suicide [11–13]. Further, experimental studies indicate weight stigma causes increased eating [14, 15] and is linked to disordered eating behaviors and avoidance of physical activity, creating a harmful cycle that can promote weight gain and further stigmatization [3, 16]. Substantial evidence suggests medical professionals display high levels of weight stigma [17–19], worsening health outcomes of patients with obesity and discouraging them from seeking care, deepening health disparities [20].

### Goals of Current Study

The prevalence of weight stigma and its negative effects have driven efforts aimed at preventing and reducing it. Research in this area is expanding rapidly, with novel approaches being tested and implemented worldwide. In light of this progress, synthesizing the latest findings on effective strategies is essential. Previous reviews have made important contributions but were published nearly a decade ago or limited to specific subpopulations (e.g., fitness professionals, healthcare students) [21–25]. Therefore, this systematic review aims to evaluate the existing strategies for reducing weight bias across all populations, including children, and identify promising directions for future research.

Moreover, weight stigma can be expressed both externally and experienced internally [26]. External weight stigma involves behaviors such as experiencing verbal abuse, exclusion, and unequal treatment based on body weight. Internalized weight stigma occurs when societal stereotypes about weight are internalized, leading to negative self-perception and self-blame. As both are important aspects of weight stigma, we examine each as an outcome.

## Methodology

### Search Strategy

This study was pre-registered on PROSPERO (CRD42024591863) and performed according to the Preferred Reporting Items for Systematic Review and Meta-Analysis (PRISMA) statement. Two authors (CW/WM) conducted a comprehensive search across multiple databases, including PubMed/Medline, PsycINFO, and Google Scholar. We also searched the reference lists of relevant articles and reviews. Iterations of the following keywords were searched using various Boolean operators: “anti-fat,” “fat,” “obesity,” “weight,” “attitude,” “bias,” “discrimination,” “prejudice,” “stereotype,” “stigma,” “alter,” “change,” “decrease,” “intervention,” “modification,” “prevention,” “reduction,” and “strategy.” This search was limited to articles published in English between the inception of the databases and October 2024.

### Study Selection

**Fig. 1:**
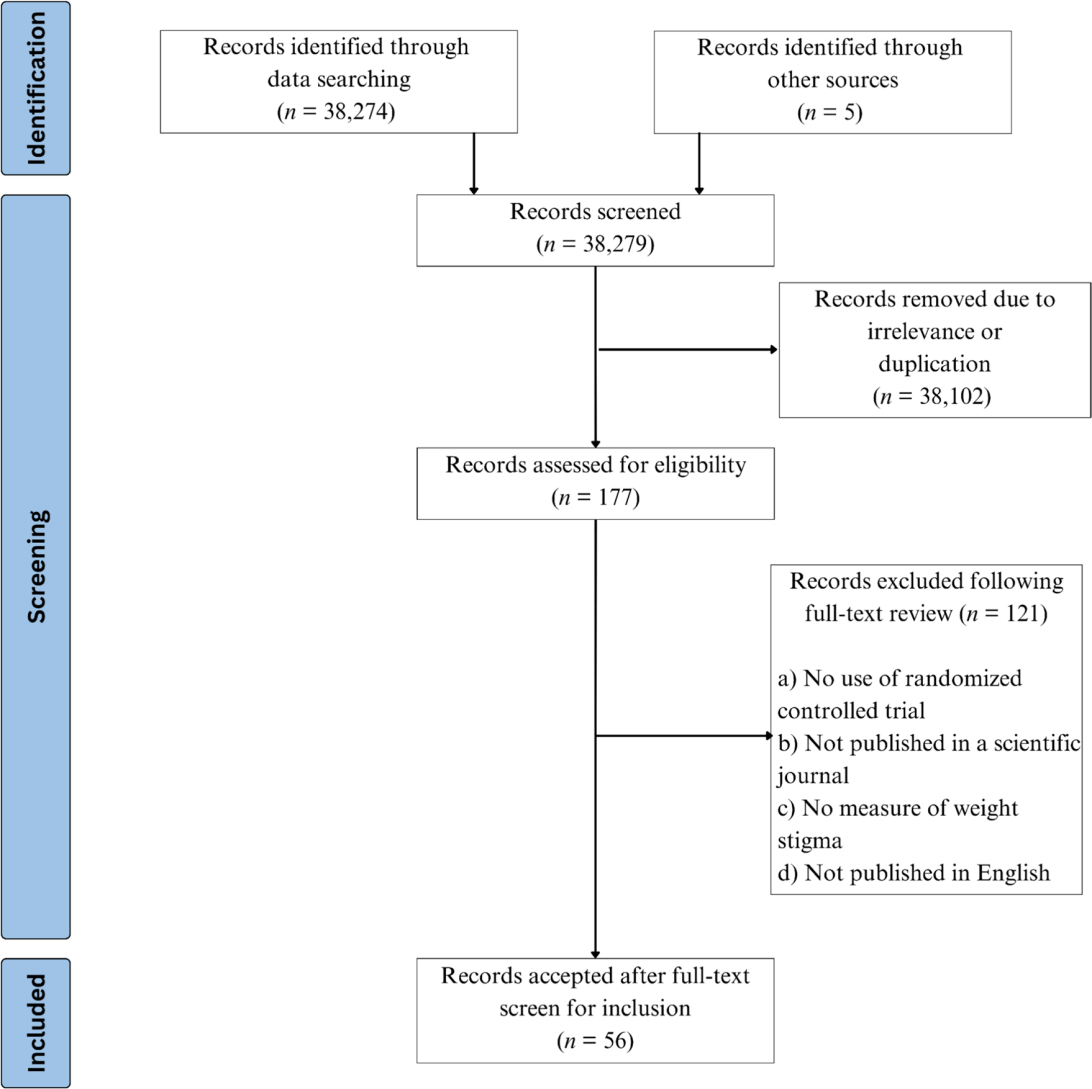
PRISMA flow diagram depicting studies retrieved and included

This review exclusively examines randomized controlled trials (RCTs) aimed at reducing weight stigma. Inclusion eligibility included studies (a) with an RCT design, (b) published in scientific journals, (c) using a validated quantitative measure for weight stigma (e.g., the Anti-Fat Attitudes Questionnaire, the Weight Bias Internalization Scale), and (d) published in English. Papers offering suggestions to combat weight stigma but without original data (e.g., review studies, policy papers) were excluded. Similarly, theses/dissertations and conference abstracts/submissions were excluded. Two authors (CW/WM) independently screened the full list of 38,279 articles to provide initial eligibility decisions by reviewing titles, abstracts, and full text. Figure 1 displays the PRISMA selection chart.

### Extraction

Two authors (CW/WM) independently assessed the articles to extract data using a standardized data extraction form. The form included study and population characteristics, and intervention details and outcomes. Discrepancies were resolved through discussion and consultation with the senior author (AJT). Narrative synthesis identified and analyzed recurring themes.

### Quality Assessment

Searching multiple databases mitigated the risk of coverage bias. To assess each study’s risk of bias, we used the Study Quality Assessment Tools developed by the National Heart, Lung, and Blood Institute from the National Institutes of Health (NIH), which rates study quality (e.g., “Was a sample size justification, power description, or variance and effect estimates provided?” “Was the loss to follow-up after baseline 20% or less?” etc.). Each study was assessed by two independent raters (CW with SP/CS) and given a rating of “Poor,” “Fair,” or “Good” quality. Disagreements were resolved through discussion and consultation with the senior author (AJT).

## Results

### Summary of Findings

Among 38,274 articles, 56 RCTs met the inclusion criteria. Of these, 22 focused on adult populations, 16 on college students, 15 on healthcare and wellness professionals or students, two on children, and one on college students and adults in separate studies. A summary of the studies is presented in Table 1. A total of 23 RCTs were rated as poor, 21 as fair, and 12 as good (Supplemental Table S1). A narrative analysis revealed eight distinct strategies: 1) shifting causal attributions and reduction of controllability beliefs, 2) weight-inclusive frameworks, 3) empathy evocation, 4) cognitive dissonance, 5) building a connection with individuals with higher weight, 6) education on caring for individuals with higher weight, and 7) multiple methodologies. The results for the identified themes are discussed below.

**Table 1:** Summary of Randomized Controlled Trial Study Characteristics

Study	Design	Population	Country	Intervention	Stigma Measure	Main Findings	NIH Quality
Alleva et al. (2021)	RCT	*N* = 98 adult women	Netherlands	Writing exercise (15 min) where participants wrote about what the body of a larger woman can do	Fat Attitudes Assessment Toolkit [External]	The intervention group reported higher levels of weight acceptance compared to the control group	Good
Berry & Myrne (2021)	RCT	*N* = 308 adults	UK, US, Canada, New Zealand, and Australia	Brief cartoon (< 1 min) from Obesity Canada's"Bust the Bias"series	Implicit Association Tasks; 14-item Fat Phobia Scale [External]	No differences between conditions	Poor
Besharatifar et al. (2024)	RCT-C	*N* = 124 adolescents	Iran	Lecture (60 min) and discussion (120 min) concerning causes of higher-weight	Attitude Toward Obese Persons Scale; Beliefs About Obese Persons Scale [External]	No differences between conditions	Fair
Braun et al. (2022)	RCT	*N* = 28 adult women with class 3 obesity	US	Eight weekly sessions (2 h) of guided mindful self-compassion meditation and discussion	Weight Bias Internalization Scale; 12‐item Weight‐Self Stigma Questionnaire [Internal]	The intervention group reported reduced weight bias internalization compared to the control group	Fair
Breithaupt et al. (2020)	RCT	*N* = 156 college students	US	Telling participants that they had anti-fat attitudes that were inconsistent with their values	Implicit Association Tasks; Anti-Fat Attitudes Questionnaire [External]	The intervention group reported reduced explicit, but not implicit, weight bias compared to the control group	Fair
Brochu et al. (2020)	RCT	*N* = 225 adults	US	A brief essay (278 words) about weight relations in the United States that emphasized a group identity as Americans	Universal Measure of Bias—Negative Judgement, Social Distance, and Attraction subscales [External]	The intervention group reported reduced weight bias in all subscales, except for attraction, compared to the control group	Fair
Burmeister et al. (2017)	RCT	*N* = 109 college students	US	A segment (17 min) of HBO's"The Weight of the Nation"docuseries discussing prejudice against people with obesity	Universal Measure of Bias—Negative Judgement, Social Distance, Attraction, and Equal Rights subscales [External]	The intervention group reported reduced weight bias in all subscales, except for attraction, compared to the control group	Good
Cha et al. (2022)	RCT	*N* = 178 adults	US	40 photos of a racially diverse higher-weight woman working out (15 min)	Fat Phobia Scale Short Form [External]	The intervention group reported reduced weight bias compared to the control group	Fair
Ciao & Latner (2011)	RCT	*N* = 64 college students	US	Telling participants that they had anti-fat attitudes that were inconsistent with their values	Anti-Fat Attitudes Test [External]	The intervention group reported reduced anti-fat attitudes (total, Physical/Romantic Unattractiveness subscale, and social/character disparagement subscale) compared to the control group	Good
Crerand et al. (2007)	RCT	*N* = 123 adult women who are obese	US	Information about the causes of obesity, the role of weight in self-esteem, and weight-related discrimination and bias, as well as strategies to improve self-esteem, body image, and quality of life	Attitude Toward Obese Persons Scale; Beliefs About Obese Persons Scale [External]	The intervention group reported reduced negative weight-biased attitudes and beliefs compared to the control group at weeks 20 and 40	Poor
Davies et al. (2022)	RCT	*N* = 135 adult women	US	Three short videos (< 5 min) of a woman expressing gratitude for her body functions and a brief creative writing exercise (10 min)	Weight Bias Internalization Scale—Modified [Internal]	The intervention group reported reduced weight bias internalization compared to the control group	Poor
Dunaev et al. (2018)	RCT	*N* = 329 adults	US	Asking participants to imagine interacting with a "confident/attractive obese person" (counter-stereotypic) and write a short response	Anti-Fat Attitudes Questionnaire—Dislike subscale modified; Universal Measure of Bias—Negative Judgement, Social Distance, and Attraction subscales; Fat Phobia Scale—Short Form [External]	The intervention group reported reduced weight bias (dislike, negative judgment, and social distance subscales) compared to the other groups	Fair
Fitzgerald et al. (2013)	RCT	*N* = 176 children	Ireland	Photo of a higher-weight girl with the cause of higher weight presented as either biological, environmental or no cause	Adjective Checklist, Shared Activity Questionnaires [External]	Biological and control condition endorsed more favorable ratings than environmental	Fair
Fogaca et al. (2024)	RCT	*N* = 46 professionals in the healthcare industry	US	Online course (2 h) discussing weight inclusivity, health enhancement, respectful care, eating for well-being, and life-enhancing movement	Fat Attitudes Assessment Toolkit [External]	The intervention group reported reduced weight bias (total and fat acceptance subscale) compared to the control group	Fair
Frederick et al. (2016)	RCT	*N* = 2187 adults	US	Constructed news articles framing fatness as negative (unhealthy, controllable, acceptable to stigmatize) or positive (healthy, uncontrollable, unacceptable to stigmatize)	Anti-Fat Attitudes Questionnaire – Willpower subscale; Anti-fat Attitudes Scale [External]	The fat-positive framing group expressed less anti-fat prejudice, willingness to discriminate against fat people, and less willingness to celebrate body-size diversity compared to the fat-negative framing group	Poor
Frederick et al. (2016)	RCT	N1 = 99 college students N2 = 114 college students N3 = 293 college students	US	News articles presented high body weight according to one or more of the following frames: 1) public health crisis; 2) personal responsibility; 3) health at every size (HAES); or 4) fat rights	Anti-fat Attitudes Scale; 3-item willingness to celebrate body size diversity [External]	Fat Rights framing group reported fewer anti-fat attitudes and more willingness to celebrate body-size diversity compared to other groups	Poor
Gloor & Puhl (2016)	RCT	*N* = 650 adults	US	A first-person narrative of an individual with obesity (Empathy), wrote as if they were an individual with obesity (Perspective Taking), read a short essay about causes of obesity (Causal), or read a shortened combined version of Empathy and Causal (Hybrid)	Fat Phobia Scale Short Form, Social Distance, Affective Reactions [External]	No differences between conditions	Poor
Hague & White (2005)	RCT	*N* = 258 adults	US	A self-paced educational module to facilitate meaningful learning (< 5 h)	Anti-Fat Attitudes Test [External]	The intervention group reported reduced anti-fat attitude compared to the control group	Poor
Haley et al. (2024)	RCT	*N* = 34 adult obese women	US	Three weekly self-compassion sessions (90 min) of psychoeducation, group discussion, experiential exercises, and assigned home practice	Weight Bias Internalization Scale [Internal]	The intervention group reported reduced weight bias internalization compared to the control group	Fair
Harris et al. (1991)	RCT	*N* = 244 college students	US	An interview with an obesity expert discussing causes/attitudes related to obesity and interviews with either high-status overweight persons or likable overweight persons	18 adjectives for individuals who are “substantially overweight” [External]	No differences between conditions	Poor
Hilbert (2016)	RCT	N1 = 128 college students N2 = 128 adults	Germany	Interactive audio-visual slide show (60 min) on weight controllability	Anti-Fat Attitudes Test; Beliefs About Obese Persons Scale; Implicit Association Tasks [External]	The college student intervention group reported reduced explicit, but not implicit weight bias compared to the control group	Poor
Huelleman et al. (2023)	RCT	*N* = 254 college women	Canada	Five days of thinking/writing exercises where participants described a time they felt bad about their body, and then reframed it with self-kindness, common humanity, and mindfulness	13-item Two-Factor Weight Bias Internalization Scale [Internal]	No differences between conditions	Good
Joseph & Raque (2023)	RCT	*N* = 189 healthcare students	US	Guided loving-kindness meditation session (10 min) with a photo of a higher-weight woman	Implicit Association Tasks; Attitude Toward Obese Persons Scale [External]	No differences between conditions	Fair
Koball & Carels (2015)	RCT	*N* = 156 college students	US	9-min direct in-lab interaction with a confederate who was obese, 1-min imagined contact with a stranger, or 3-min video of positive interpersonal interaction between two friends (one obese, one normal weight)	Obese Person Trait Survey; Anti-Fat Attitudes Questionnaire—Dislike subscale; Behavioral intentions [four questions modified from Ratcliff et al. (1999)] [External]	The direct contact group reported reduced weight bias and increased behavioral intentions to interact with obese persons	Poor
Kramer et al. (2024)	RCT	*N* = 197 female college students	US	Prescribing advice to hypothetical pre-teenage girls inquiring about body image issues from their personal experience or arguing against legal policies discriminating against higher-weight folks	Anti-Fat Attitudes Test; Weight Bias Internalization Scale – Modified; Goldfarb Fear of Fat Scale; Implicit Association Tasks [Both external and internal]	The intervention groups reported reduced anti-fat attitude and weight bias internalization compared to the control group; Only the group responding from their own experience reported reduced implicit weight bias	Fair
Kreynin et al. (2024)	RCT	*N* = 67 healthcare students	US	Two weekly (2 h) group sessions aimed at critically examining weight stigma in health professional students’ personal and professional lives	Goldfarb Fear of Fat Scale; Ideal Body Stereotype Scale—Revised; Universal Measure of Bias; Eating Pathology Symptoms Inventory – Negative Attitudes Towards Obesity Subscale [External]	The intervention group reported reduced weight bias internalization and weight bias compared to the control group. Reduction in weight bias (but not anti-fat subscale in Universal Measure of Bias) sustained at the four-week follow-up	Poor
Ksinan et al. (2017)	RCT	*N* = 580 adults	Czech Republic	A one-page scientific article about the topic of health focusing on either willpower or genetics as causes of obesity	Anti-Fat Attitudes Questionnaire—Translated [External]	Willpower condition participants reported higher, and Genetics condition participants reported lower anti-fat attitude compared to the control group	Fair
Lee et al. (2024)	RCT	N1 = 344 college students N2 = 168 college students	US	Study 1: Asked participant to write a message to their recipient (close other or stranger) showing concern about the stigmatizing scenario and wishing them health, happiness, and wellbeing (3 min) Study 2: asked participant to write a message to close other or write a message about the situation and give support	Fat Phobia Scale—Short Form; Implicit Association Tasks [External]	No differences between conditions	Fair
Lin & Stutts (2020)	RCT	*N* = 350 adults	US	Exposed participants to four persons with obesity due to either controllable or uncontrollable factors	Fat Phobia Scale—Short Form; Beliefs About Obese Persons Scale [External]	The uncontrollable factors group reported reduced weight bias compared to the controllable factors group	Fair
Lopez-Lara et al. (2024)	RCT	*N* = 242 healthcare students	Mexico	A lecture (60 min) concentrated on body weight regulation and weight stigma, highlighting its consequences in healthcare	Fat Phobia Scale—Short Form; Beliefs About Obese Persons Scale [External]	The intervention group reported reduced negative weight-biased beliefs compared to the control group	Poor
Martingano et al. (2023)	RCT	*N* = 582 adults	US	Short educational video (5 min) explaining how a person's environment can interact with their genetics to influence eating behaviors	Anti-Fat Attitudes Questionnaire [External]	The intervention group reported less blame toward people with higher weight (measured by willpower subscale) compared to the control group	Fair
Matharu et al. (2014)	RCT	*N* = 129 healthcare students	US	Dramatic reading (60 min) of "The Most Massive Woman Wins" play script followed by a group discussion	Anti-Fat Attitudes Questionnaire; Implicit Association Tasks [External]	The intervention group reported reduced explicit, but not implicit weight bias compared to the control group	Good
Myre et al. (2020)	RCT	*N* = 103 adult women	Canada	Three weekly sessions of viewing counter-stereotypical images of active individuals with obesity paired with positive physical activity-related words	Weight Bias Internalization Scale—Modified [Internal]	The intervention group reported reduced weight bias internalization compared to the control group. The effect was maintained at a one-week follow-up	Fair
Nickel et al. (2019)	RCT	*N* = 949 adults	Germany	Animated video (2.5 min) providing neutral information on obesity and obesity treatment	Fat Phobia Scale [External]	No differences between conditions	Poor
Nutter et al. (2018)	RCT	*N* = 309 adults	US, Canada	A brief article stating obesity is or is not a disease	20-item Universal Measure of Bias Fat [External]	Obesity-disease condition reported reduced weight bias compared to other conditions	Fair
O'Brien et al. (2010)	RCT	*N* = 159 healthcare students	UK	Three weekly tutorials where participants read a short passage about common controllable and uncontrollable causes of obesity	Anti-Fat Attitudes Questionnaire; Belief About Obese Persons Scale; Implicit Association Tasks [External]	The uncontrollable factors group reported reduced implicit and explicit anti-fat attitudes compared to other groups	Poor
O'Brien et al. (2020)	RCT	N1 = 530 college students N2 = 690 college students	Australia	A newspaper article about mortality from obesity-related diseases focusing on either food addiction or exercise	Anti-Fat Attitudes Test [External]	The food addiction group reported reduced anti-fat attitude compared to other groups	Poor
Oliver et al. (2022)	RCT-C	*N* = 99 healthcare students	US	Three sessions (1 h) with information about the prevalence of obesity, genetic influences related to obesity, the presence and negative impact of weight bias within healthcare	Attitude Toward Obese Persons Scale; Beliefs About Obese Persons Scale [External]	The intervention group reported reduced weight-biased beliefs, but not attitudes, compared to the control group	Fair
Palmeira et al. (2017)	RCT	*N* = 73 adult women who are obese	Portugal	Ten weekly group sessions promoting mindfulness, acceptance, and compassion plus two booster sessions (2.5 h in total)	Weight Self-Stigma Questionnaire [Internal]	The intervention group reported reduced weight bias internalization compared to the control group	Good
Pearl et al. (2020)	RCT	*N* = 72 adults who are obese	US	Twelve 90-min weekly group sessions, followed by two every-other-week sessions and two monthly sessions (16 sessions over 26 weeks total), as well as 30-min stigma reduction focused on weight myths and self-acceptance	Weight Bias Internalization Scale; Weight Self-Stigma Questionnaire; Fat Phobia Scale [Both external and internal]	The intervention group reported reduced weight bias internalization (measured by Weight Self-Stigma Questionnaire; total and self-devaluation subscale) compared to the control group at weeks 12 and 26	Good
Pearl et al. (2023)	RCT	*N* = 105 adults who are obese	US	Twenty weekly group sessions, followed by six monthly sessions and three every-other-month sessions plus additional education on weight stigma’s impacts and ways to overcome the stigma (90 min per session)	10-item Weight Bias Internalization Scale; Weight Self-Stigma Questionnaire [Internal]	The intervention group reported reduced weight bias internalization (measured by Weight Self-Stigma Questionnaire) compared to the control group at Week 46	Good
Persky et al. (2011)	RCT	*N* = 110 healthcare students	US	A short article explaining either genetics or behavior to be the cause of obesity	Obese Person Trait Survey [External]	The genetics group reported reduced stereotyping compared to control group	Poor
Potts et al. (2022)	RCT	*N* = 55 adults who are obese	US	One chapter of “The Diet Trap” self-help book per week and received either phone coaching or email check-ins	Weight Self Stigma Questionnaire [Internal]	The intervention groups reported reduced weight bias internalization compared to the control group	Fair
Rodriguez et al. (2016)	RCT	*N* = 109 college students	US	Asked participant to walk across campus wearing a fat suit	Anti-Fat Attitudes Questionnaire—Dislike subscale [External]	No differences between conditions	Good
Rosenbaum (2024)	RCT	*N* = 319 college students	US	A third-person narrative focused on a fictional 22-year-old college student with obesity	Anti-Fat Attitudes Test [External]	The intervention groups reported reduced weight bias (total and controllability subscale) compared to the control group	Fair
Rudolph & Hilbert (2017)	RCT	*N* = 506 (baseline), 230 (7-day follow-up), 145 (30-day follow-up) healthcare professionals	Germany	Information on making behavioral changes to enhance health	The Beliefs About Obese Persons Scale; The Attitudes About Obese Persons Scale; The Affective Priming Task; Implicit Association Tasks [External]	The intervention groups reported reduced implicit, but not explicit, weight bias compared to the control group	Poor
Sherf-Dagan et al. (2022)	RCT	*N* = 162 (baseline), 152 (1-week follow-up), and 146 (6-week follow-up) healthcare professionals	Israel	Online educational module (15 min) discussing obesity causes and stigma	Anti-Fat Attitudes Questionnaire; Fat Phobia Scale [External]	The intervention groups reported reduced weight bias (measured by Fat Phobia Scale) compared to the control group	Good
Sherf-Dagan et al. (2024)	RCT	*N* = 162 (baseline), 152 (1-week follow-up), and 146 (6-week follow-up) healthcare professionals	Israel	Three short video lectures covering knowledge, stigma, and strategies related to weight	Anti-Fat Attitudes Questionnaire; Fat Phobia Scale—Short; Implicit Association Tasks [External]	The intervention groups reported reduced both explicit and implicit weight bias compared to the control group	Good
Speirs et al. (2022)	RCT	*N* = 249 professionals in the healthcare industry	New Zealand	A short article explaining calories, disease, or food addiction as causes of obesity	13-item Anti-Fat Attitudes Questionnaire [External]	The food addiction group reported reduced weight bias compared to the disease group	Poor
Swift et al. (2013)	RCT	*N* = 43 healthcare students	UK	Two short Rudd Center films (17 min each) titled “Weight Prejudice: Myths and Facts” and “Weight Bias in Healthcare” about causes of obesity and raising awareness of bias in healthcare	Fat Phobia Scale; Beliefs About Obese People Scale; Implicit Association Tasks; Anti-Fat Attitudes Questionnaire—Dislike and Willpower subscales [External]	The intervention groups reported reduced explicit, but not implicit, weight bias compared to the control group	Fair
Turner et al. (2012)	RCT	N1 = 60 college students N2 = 50 college students	UK	Asked participants to spend 5 min imagining a nostalgic interaction with an obese person	A composite score of feelings, beliefs, behavioral intentions toward overweight persons (5 items each) [External]	The intervention groups reported reduced weight bias compared to the control group	Poor
Turner et al. (2022)	RCT	*N* = 125 college students who are overweight	UK	Asked participants to spend 5 min imagining a nostalgic interaction with an obese person	Feelings, beliefs, behavioral intentions toward overweight persons (5 items each) [External]	The intervention groups reported reduced negative feelings and beliefs and increased behavioral intentions toward persons who are overweight compared to the control group	Poor
Wiese et al. (1992)	RCT	*N* = 75 healthcare students	US	A video tape of an interview with an obese nurse and her experience (2 h), a written message from a current special on the causes of obesity, and a 10-min role-play exercise concentrating on an obese female friend and her thoughts and feelings in a given situation	19-item attitudes toward obesity [External]	The intervention groups reported reduced negative attitudes toward obesity after five weeks compared to the control group. The effects for reduced blaming and perceiving obesity as controllable sustained after one year	Poor
Wijayatunga et al. (2021)	RCT	*N* = 147 professionals in the healthcare industry	US	A 20-min video that highlights the complex nature of obesity's etiology and the negative impact of communication styles and induces empathy	Anti-Fat Attitude Test (AFAT); Implicit Association Tasks [External]	No differences between conditions	Good
Wilson et al. (2020)	RCT	*N* = 94 female college students	US	Two 90–120 min interactive group sessions designed to engender the rejection of dieting, increase body acceptance, and develop healthy eating skills	The Anti-Fat Attitudes Questionnaire [External]	The intervention groups reported reduced weight bias compared to the control group, and the effect was sustained after one month	Poor
Zuest et al. (2024)	RCT	*N* = 105 professionals in healthcare industry	US	Online course (2 h) discussing weight inclusivity, health enhancement, respectful care, eating for well-being, and life-enhancing movement	Fat Attitudes Assessment Toolkit [External]	The intervention groups reported reduced weight bias compared to the control group	Poor

#### Shifting Causal Attributions and Reducing Controllability Beliefs

Weight stigma can stem from the misconception that obesity is solely due to individual, controllable factors such as laziness or lack of discipline [27]. Some studies highlighted the impact of genetics, biology, and environmental influences on dismantling negative stereotypes [28]. We identified 15 articles that presented participants with scientific evidence on the complex nature of obesity, with two articles conducting two studies each [29–43]. All studies targeted external weight bias with varying results.

Nine studies employed this strategy to reduce weight bias among general adults, with seven reporting success [29, 31, 33–38, 42]. Among these, five used written materials, one used a five-minute educational video, and another invited individuals with obesity to discuss the causes of obesity. Two studies did not observe changes in weight-biased attitudes. Hilbert had participants view an hour-long interactive slideshow on weight controllability, which did not change stigmatizing attitudes relative to the control group [33]. Similarly, Berry and Myrne’s study using Obesity Canada’s ‘Bust the Bias’ short video, which explained that exercise may not impact body weight, had no impact on explicit or implicit bias, measured by the Fat Phobia Scale and the Implicit Association Test (IAT), respectively [29].

Interventions among college students yielded more consistent success, with three studies reporting at least partial improvements [32, 33, 40]. Frederick et al. found that college students who read articles challenging the controllability of fatness and condemning weight discrimination reported lower anti-fat attitudes and greater acceptance of size diversity [32]. O’Brien et al. conducted two experiments with college students, providing the intervention groups with either a food addiction or a diet and exercise explanation for obesity [40]. In the first experiment, students who received the food addiction explanation showed reduced anti-fat attitudes, particularly in the dislike subscale, which measures explicit prejudice toward higher body weight, and the willpower subscale, which assesses the belief that obesity results from a lack of self-discipline. However, in the second experiment, the decrease was limited to the dislike subscale, and the intervention group showed no significant difference from the control group. Hilbert’s intervention, although ineffective among their adult sample, successfully reduced explicit weight stigma among college students [33].

Three studies focusing on healthcare workers or students demonstrated changes in their participants’ weight-biased attitudes [39, 41, 43]. Persky et al. found that students who learned about the genetic mechanisms of obesity reported less negative stereotyping than those who read about a control topic [41]. O’Brien et al. found that healthcare students who learned about uncontrollable reasons for obesity reported reduced implicit anti-fat attitudes compared to those who learned about controllable causes or an unrelated health topic (control). Although intervention participants reported decreased dislike, explicit anti-fat prejudice did not differ across groups [39]. Speirs et al. reported that practitioners who received a food addiction explanation for obesity had lower anti-fat attitudes on the willpower subscale but not the dislike subscale compared to those who received a disease explanation [43].

Lastly, one study targeted elementary children [30]. Although children who read about an uncontrollable cause (biology) reported more positive attitudes toward an overweight peer than a controllable cause, overall group differences in attitudes were not significant.

#### Weight-Inclusive Initiatives

Weight-inclusive initiatives focus on body acceptance, self-compassion, and the promotion of health and well-being independent of body size, encouraging individuals to adopt healthy behaviors without emphasizing weight loss. Eleven studies employed weight-inclusive initiatives [44–54]. Seven of these studies focused on adults [44–48, 51, 52], with five comprising women [44, 45, 47, 48, 51]. Alleva et al. and Cha et al. reduced external weight bias by asking participants to write about the capabilities of a larger woman’s body and by displaying photos of higher-weight women exercising [44, 46]. Notably, we rated Alleva et al.’s study as good quality based on the NIH assessment. The remaining studies focused on internalized weight bias. Three studies implemented weekly sessions centered on self-compassion and mindfulness [45, 48, 51]. One study combined short videos of a woman expressing gratitude for her body’s functions [47], and another study asked participants to read a self-help book chapter about acceptance and commitment therapy [52].

Three studies with college students reported mixed results with weight-inclusive interventions [49, 50, 54]. Two studies focused on external weight bias. Wilson et al. reported a reduction in anti-fat attitudes among participants who attended sessions on increasing body acceptance and developing healthy dieting behaviors, with the effects sustaining after one month [54]. However, Lee et al. found that loving-kindness meditation, intended to foster compassion for oneself and others, failed to reduce fat-phobia bias [50]. Similarly, Huelleman et al., whose study was rated as good quality per the NIH assessment, found differences in internalized weight bias between participants who engaged in self-kindness and mindfulness writing exercises and those who wrote about appearance ideals for women or did not complete any writing exercises [49].

The final study targeting healthcare students also employed loving-kindness meditation. Using the Attitude Toward Obese Persons Scale and the IAT to measure explicit and implicit weight bias, respectively, found no differences in explicit or implicit weight stigma compared to the control group [53].

#### Inducing Empathy

Empathy-inducing interventions included narratives, personal testimonials, or immersive activities designed to help participants understand weight stigma’s emotional and psychological impact [55]. Two studies targeted external weight stigma [56, 57], and both rated good quality per the NIH assessment. Rodriguez et al. had college students experience life with higher weight by walking across the campus wearing a fat suit, but this did not significantly lower anti-fat attitudes compared to controls [56]. Matharu et al. focused on healthcare students and reported partial success in changing participants’ weight-biased attitudes [57]. Their intervention involved a 60-minute dramatic reading of *The Most Massive Woman Wins* and a group discussion. Participants in the intervention group reported reduced anti-fat attitudes compared to the control group. However, there were no differences in implicit weight bias across groups.

#### Cognitive Dissonance

Festinger’s cognitive dissonance theory suggests that inconsistencies between beliefs, attitudes, and actions create psychological discomfort, motivating individuals to resolve the discrepancy by adjusting their beliefs and attitudes [58]. Three studies examined cognitive dissonance as a strategy for preventing weight stigma among college students [59–61]. Two studies focused on external weight bias. One, rated as good quality per the NIH assessment, presented participants with either a cognitive dissonance intervention, highlighting inconsistencies between their attitudes and values of kindness and equality, or a social consensus intervention, suggesting their views were more stigmatizing than their peers [59]. The cognitive dissonance intervention reduced explicit anti-fat attitudes, whereas the social consensus intervention showed no difference from controls. Another study employed a similar cognitive dissonance strategy [60]. They found that, at the one-week follow-up, intervention participants reported lower explicit anti-fat attitudes than the control group, but implicit attitudes remained unchanged. Lastly, Kramer et al. explored cognitive dissonance through two writing-based interventions aimed at addressing both external and internal weight bias [61]. These writing interventions required participants to either give advice to hypothetical pre-teenage girls about body image based on personal experience or argue against legal policies discriminating against individuals with higher weight. Both interventions reduced explicit anti-fat bias compared to the control, but only the former led to reductions in implicit bias.

#### Building Connection

Interventions designed to build connections emphasize shared humanity, values, and positive contact with “outgroup” individuals. This category of intervention includes the induction of “a sentimental longing or wistful affection for the past” to increase social connections and thereby reduce weight stigma. Six studies employed interventions under this category [62–67]. Three studies targeted adults. Brochu et al. and Dunaev et al. targeted external weight bias and found reduced weight bias attitudes in the intervention groups compared to the control groups. In Brochu et al.’s study, intervention participants read about weight-related issues in the United States, emphasizing a shared identity as Americans [64]. These participants reported lower levels of weight bias compared to control participants. In Dunaev et al.’s study, intervention participants who imagined interacting with a confident and attractive person with obesity reported lower levels of weight bias than those who imagined interacting with an insecure and unattractive person with obesity [65]. Myre et al. addressed internal weight bias, focusing solely on female participants [67]. They exposed intervention group participants to images of active individuals with obesity paired with positive words related to physical activity. Women in the intervention group reported reduced internalized weight bias, and this effect persisted for one week.

The remaining three focused on college students. Koball and Carel’s study reported reduced external weight bias [66]. Intervention participants who engaged in direct interaction with a confederate with obesity exhibited lower weight bias and greater behavioral intentions to interact with individuals with obesity compared to those in the control group and who watched a video of a positive interaction between friends of mixed weight status. The other two studies were conducted by Turner et al. Both involved asking participants to imagine a sentimental, nostalgic interaction with a higher-weight individual [62, 63]. In two samples, participants in the nostalgic interaction condition exhibited more positive attitudes toward higher-weight individuals compared to the control [62]. Similarly, the second study found that the nostalgia intervention led to more positive attitudes than the control [63].

#### Providing Education on Caring for Individuals with Obesity

Educational interventions were also used. These aimed to challenge stereotypes surrounding weight stigma, provide evidence-based information, and teach techniques to counteract weight bias. Three studies used this strategy [68–70]. Hague and White targeted the general adult population with an educational module covering the causes of obesity, the negative effects of weight stigma, and strategies to address it [68]. Participants reported reduced anti-fat attitudes after the intervention, with effects sustained for six weeks.

Two studies focused on reducing external weight stigma by increasing education on caring for larger individuals among healthcare professionals and students. Sherf-Dagan et al. developed a good-quality study, as rated by the NIH assessment, of a 15-minute online educational module covering obesity, the definition and impact of weight bias, strategies for reducing weight bias, and a quiz [69]. The intervention decreased fat-phobic attitudes over 30 days, though there were no changes in anti-fat attitudes between groups over time. Oliver et al. designed a longer educational intervention for healthcare students, covering obesity prevalence, the impact of weight bias in healthcare, and strategies for reducing bias in clinical settings [70]. Beyond watching and discussing a video on weight bias and completing reflective journaling exercises, the intervention group received two additional training sessions, personalized feedback on their weight bias scores, case-based learning scenarios requiring critical thinking, and a group discussion on their reflections. The intervention group reported more favorable beliefs about individuals with obesity but not in their attitudes toward individuals with obesity compared to the control group.

#### Combining Multiple Approaches

Sixteen studies employed interventions that combined two or more strategies [71–86]. Four of these studies focused on adults [73, 75, 79, 80], with three targeting external weight bias and reporting mixed results in bias reduction. Crerand et al. provided participants with information about obesity causes and self-esteem improvement techniques [73]. Participants in the intervention group reported less negative attitudes and less negative beliefs about obesity at weeks 20 and 40. Gloor and Puhl found no differences between the intervention and control groups [75]. Their participants engaged in activities such as reading a first-person narrative about an individual with obesity, imagining themselves as an individual with obesity, reading about the causes of obesity, or reading a combined version of the first-person narrative and causes of obesity. However, none of the intervention groups reported reduced weight bias.

Pearl et al. conducted two good-quality studies per the NIH assessment targeting internalized weight bias. Intervention participants in the 2020 study attended sessions on obesity causes, self-acceptance, and psychoeducation about weight bias and its effects. Participants reported improvements in the weight self-stigma total score and the self-devaluation subscale in the 12 th week and sustained in week 26 [79]. The 2023 study employed a similar intervention, with all participants receiving behavioral weight loss sessions [80]. The intervention group received additional training on obesity, weight bias, and self-acceptance. The intervention group reported greater improvements in weight self-stigma by week 46.

Three studies of college students and external weight stigma employed varied methods with mixed effects on attitudes [72, 76, 81]. Burmeister et al., whose study was rated as good quality per the NIH assessment, combined educational information with empathy induction by showing participants the weight stigma section of Home Box Office (HBO)’s *The Weight of the Nation* documentary [72]. The intervention group reduced their negative attitudes toward individuals with obesity. In Rosenbaum’s study [81], participants in the intervention group read a fictional narrative about a college student with obesity, intended to reduce weight controllability beliefs and elicit empathy. These participants reported reduced overall weight bias, sustained at one-month follow-up, but no differences in stereotype-related attitudes or perceptions of unattractiveness. Harris et al. [76] showed an expert interview on the causes and treatment of obesity and social attitudes toward individuals with obesity, followed by an interview with an individual with obesity. However, the intervention did not change weight-biased attitudes compared to the control groups.

Most studies using combined methods targeted healthcare and wellness professionals and students [74, 77, 78, 82–86]. Seven of the eight studies focused on external weight bias [74, 78, 82–86]. Fogaca et al. designed an online course for university recreation center professionals in the intervention group [74]. The course presented causal and informational content on obesity and provided strategies to reduce weight stigma. Intervention participants reported a reduction in weight-biased attitudes compared to the control group. Using the same intervention and targeting a similar population, Zuest et al. reported increases in the fat acceptance score in the intervention group [86]. Lopez-Lara et al. developed an educational session for healthcare professionals to address obesity and weight stigma while promoting a weight-inclusive healthcare environment [78]. The intervention group demonstrated reduced negative beliefs about individuals with obesity compared to the control group.

Sherf-Dagan et al. developed good-quality intervention, as rated by the NIH assessment, for healthcare students included short video lectures on obesity, weight bias, and stigma reduction, vignettes depicting interactions between health professionals and individuals with obesity, and group discussions with an individual with obesity [82]. Over six weeks, the intervention group showed reductions in anti-fat attitudes and fat phobia scores. Wiese et al. reduced healthcare students’ weight-biased attitudes by showing an interview with a nurse with obesity to induce empathy, reading about obesity causes, and completing role-playing activities [84]. The intervention group reported lower weight bias at week five and were more likely to perceive obesity as uncontrollable one year later. Swift et al. [83] had the intervention group watch two 17-minute films: *Weight Prejudice: Myths and Facts*, featuring a teenager sharing her experiences with being overweight alongside expert commentary on obesity causes, and *Weight Bias in Healthcare*, where a former supermodel and activist discussed the stigma faced by patients with obesity. Although the intervention group showed reduced explicit negative attitudes and beliefs toward obese individuals, their implicit anti-fat bias remained largely unchanged. In contrast, Wijayatunga et al., whose study was rated good quality per the NIH assessment, found no changes between the intervention and control groups [85] with an intervention of a 20-minute video on the uncontrollability of obesity and role-playing.

Kreynin et al.’s study was the only study that targeted both external and internalized weight bias [77]. Their group-based, peer-led intervention included psychoeducation on obesity and weight bias, cognitive dissonance exercises, and reflection on weight stigma in participants’ lives, patient care practices, and healthcare. Compared to the control, intervention participants reported reduced internalized weight concerns and external stigma, with effects lasting four weeks. They also showed decreases in internalized anti-fat attitudes toward others, though these changes were not sustained over time.

Finally, one paper focused on middle school students failed to change weight stigmatizing attitudes between the intervention and control groups [71]. The intervention group learned about the causes of obesity and discussed feelings about people with obesity in their lives.

## Conclusion

Many weight stigma interventions have yielded promising results, with 93% of the studies indicating lower weight stigma in the intervention group compared to the control group. However, the success rate may be inflated due to reporting bias, as null or negative findings are less likely to be published. Building connections and using cognitive dissonance reflect an expansion of strategies compared to interventions highlighted in past reviews [21, 22]. Moreover, several studies enhanced interventions by combining strategies or implementing multi-session designs, which showed greater success than single-session studies. The long-term effects of these interventions remain unknown, as discussed later.

The rigor of the studies reviewed here was relatively high, as randomized, controlled interventions allowed for causal inference and reduced bias. However, only 12 (21%) of studies were rated as “good,” based on NIH studies, partly due to missing information in their reporting. To improve future research quality, studies should report details on randomization procedures, blinding of participants and researchers, avoidance of confounding interventions, and power analyses.

A key weakness in the existing literature is the reliance on attitudinal measures as outcomes. In healthcare, for example, changing professionals'attitudes may not translate to better behavior and patient outcomes. Expanding target outcomes across domains would advance the field. For example, studies could assess whether an intervention for managers reduces size-based bias in hiring and promotion, while school-based interventions could examine improvements in the mental well-being of children with obesity.

Additionally, many studies reviewed did not include longitudinal designs or follow-up assessments, limiting our understanding of the sustained effects of interventions. This lack of longitudinal data is a significant gap, as weight stigma interventions with only short-term effects may be a waste of resources. Conversely, some interventions may have delayed or evolving impacts that short-term studies fail to capture. Future research should aim to incorporate follow-up periods to evaluate the durability of intervention effects and identify factors contributing to long-term success.

The use of varied and non-standardized instruments across all the studies complicates efforts to aggregate data and compare outcomes, potentially impacting the accuracy of the overall findings. Further research should identify a reliable and valid measure of weight stigma that is applicable across various contexts and populations to enhance consistency in the field, improve the clarity of research findings, and allow for more meaningful comparisons across studies. However, we recognize that weight stigma can be a multi-faceted construct, and different strategies may be more effective for some forms than others. Indeed, we observed that interventions focusing on weight-inclusive initiatives and building connections were more often used to reduce internal weight bias, whereas interventions that aim to shift causal attributions, induce empathy, and provide education were more often used to reduce external weight bias.

Furthermore, certain populations appear underrepresented among current randomized controlled trial research, with healthcare professionals and children being particularly understudied despite their critical roles as stakeholders. Weight bias can emerge in children as early as ages 9 to 11 [87, 88]. Although the RCTs in this review showed limited success in reducing children's weight bias, interventions should start early, with more appropriate and effective strategies. Further, while 18% of studies focused exclusively on women, aligning with prior research indicating greater weight stigma among women, recent findings suggest weight stigma is also prevalent among men. We recommend further research on men and studies investigating gender differences in weight stigma.

Additionally, the racial/ethnic and socioeconomic diversity of the samples was limited. Our review was also likely affected by the decision to include only articles published in English, potentially introducing bias and limiting comprehensiveness. This language restriction may partly explain why most included studies were conducted in developed Western countries, particularly the U.S., with only one study conducted in Mexico. Emerging evidence suggests that the experiences and impact of weight bias vary across cultures due to differing norms and values related to body weight [89, 90]. Special attention should be given to low- and middle-income countries—particularly those in Polynesia and Micronesia, the Caribbean, the Middle East, and North Africa—where obesity rates have drastically increased in recent decades [91].

In conclusion, the large body of work testing weight stigma interventions is encouraging as we strive toward a more just and equitable society. However, more work remains to strengthen the rigor of the science in this area. Once this field is advanced, the anticipated benefits to individuals across all areas of life—health, relationships, work, and education—are considerable.

## Key References

- Kramer EB, Pietri ES, Bryan AD. Reducing anti-fat bias toward the self and others: a randomized controlled trial. J Eat Disord. 2024;12(1):46.
- This paper examines internal and external anti-fat bias in a single study, which is a strength as both are important aspects of weight stigma.
- Sherf-Dagan S, Kessler Y, Mardy-Tilbor L, Raziel A, Sakran N, Boaz M, et al. The Effect of an Education Module to Reduce Weight Bias among Medical Centers Employees: A Randomized Controlled Trial. Obes Facts. 2022 May;15(3):384–94.
- A needed advancement in this field is examining practicing healthcare professionals. This paper comprises physicians, nurses, managerial staff, medical technicians, and other professionals within a large medical center where all 3,290 employees were invited to participate.
- Pearl RL, Wadden TA, Bach C, LaFata EM, Gautam S, Leonard S, et al. Long-term effects of an internalized weight stigma intervention: A randomized controlled trial. J Consult Clin Psychol. 2023 Jul;91(7):398.
- Long-term follow-ups are crucial to ensure that effects of weight stigma interventions are not fleeting. This study had a follow-up at 72 months post-intervention—by far the longest follow-up period of any study in this field.

## Supplementary Information

Below is the link to the electronic supplementary material.

Supplementary file1 (DOCX 44 KB)

## Data Availability

No datasets were generated or analysed during the current study.
