# Supplementary material for: Assessing Weight Stigma Interventions: A Systematic Review of Randomized Controlled Trials
Source: Curr Obes Rep. 2025 Apr 14;14(1):35. doi: 10.1007/s13679-025-00628-w (PMC11997004; doi:10.1007/s13679-025-00628-w)
Supplement: Supplementary file 1 — Supplementary file1 (DOCX 44 KB) [file 13679_2025_628_MOESM1_ESM.docx]

Supplement 1. NIH Quality Assessment

| **Criteria** | Alleva et al (2021) | Berry & Myrne (2021) | Besharatifar et al (2024) | Braun et al (2022) | Breithaupt et al (2020) | Brochu et al (2020) | Burmeister et al (2017) | Cha et al (2022) | Ciao & Latner (2011) |
| --- | --- | --- | --- | --- | --- | --- | --- | --- | --- |
| 1. Was the study described as randomized, a randomized trial, a randomized clinical trial, or an RCT? | 1 | 1 | 1 | 1 | 1 | 1 | 1 | 1 | 1 |
| 2. Was the method of randomization adequate (i.e., use of randomly generated assignment)? | CD | CD | 1 | CD | CD | CD | 1 | CD | 1 |
| 3. Was the treatment allocation concealed (so that assignments could not be predicted)? | 1 | CD | CD | CD | CD | CD | CD | CD | CD |
| 4. Were study participants and providers blinded to treatment group assignment? | 1 | CD | CD | 1 | 1 | CD | CD | CD | CD |
| 5. Were the people assessing the outcomes blinded to the participants' group assignments? | CD | CD | CD | CD | CD | CD | CD | CD | CD |
| 6. Were the groups similar at baseline on important characteristics that could affect outcomes (e.g., demographics, risk factors, co-morbid conditions)? | 1 | 1 | 1 | 1 | 1 | CD | 1 | 1 | 1 |
| 7. Was the overall drop-out rate from the study at endpoint 20% or lower of the number allocated to treatment? | 1 | 0 | 1 | 1 | 0 | 1 | 1 | 1 | 1 |
| 8. Was the differential drop-out rate (between treatment groups) at endpoint 15 percentage points or lower? | 1 | 1 | 1 | 1 | 1 | 1 | 1 | 1 | 1 |
| 9. Was there high adherence to the intervention protocols for each treatment group? | 1 | 1 | 1 | 1 | 1 | 1 | 1 | 1 | 1 |
| 10. Were other interventions avoided or similar in the groups (e.g., similar background treatments)? | CD | CD | CD | CD | CD | CD | CD | CD | CD |
| 11. Were outcomes assessed using valid and reliable measures, implemented consistently across all study participants? | 1 | 1 | 1 | 1 | 1 | 1 | 1 | 1 | 1 |
| 12. Did the authors report that the sample size was sufficiently large to be able to detect a difference in the main outcome between groups with at least 80% power? | 0 | 0 | 0 | 0 | 1 | 1 | 1 | 1 | 1 |
| 13. Were outcomes reported or subgroups analyzed prespecified (i.e., identified before analyses were conducted)? | 1 | CD | CD | CD | CD | CD | CD | CD | CD |
| 14. Were all randomized participants analyzed in the group to which they were originally assigned, i.e., did they use an intention-to-treat analysis? | CD | CD | CD | CD | CD | CD | CD | CD | CD |
| **Total Score** | 9 | 5 | 7 | 7 | 7 | 6 | 8 | 7 | 8 |
| **Percentile** | 92.86 | 29.46 | 67.86 | 67.86 | 67.86 | 49.11 | 83.93 | 67.86 | 83.93 |
| **Rating** | Good | Poor | Fair | Fair | Fair | Fair | Good | Fair | Good |
|  |  |  |  |  |  |  |  |  |  |
|  | *Note*: CD = Cannot Determine. Ratings were based on percentile and 0-33.99% were rated as “Poor, 34-67.99% as “Fair” and 68-100% as “Good.” | | | | | | | | |

| **Criteria** | Crerand et al (2007) | Davies et al (2022) | Dunaev et al (2018) | Fitzgerald et al (2013) | Fogaca et al (2024) | Frederick et al (2016) | Frederick et al (2016) | Gloor & Puhl (2016) | Hague & White (2005) |
| --- | --- | --- | --- | --- | --- | --- | --- | --- | --- |
| 1. Was the study described as randomized, a randomized trial, a randomized clinical trial, or an RCT? | 1 | 1 | 1 | 1 | 1 | 1 | 1 | 1 | 1 |
| 2. Was the method of randomization adequate (i.e., use of randomly generated assignment)? | CD | CD | CD | CD | 1 | CD | CD | 1 | CD |
| 3. Was the treatment allocation concealed (so that assignments could not be predicted)? | CD | CD | CD | CD | CD | CD | CD | CD | CD |
| 4. Were study participants and providers blinded to treatment group assignment? | CD | CD | CD | CD | CD | CD | CD | CD | CD |
| 5. Were the people assessing the outcomes blinded to the participants' group assignments? | CD | CD | CD | CD | CD | 1 | CD | CD | CD |
| 6. Were the groups similar at baseline on important characteristics that could affect outcomes (e.g., demographics, risk factors, co-morbid conditions)? | 1 | 1 | 1 | 1 | 1 | CD | CD | CD | 1 |
| 7. Was the overall drop-out rate from the study at endpoint 20% or lower of the number allocated to treatment? | CD | 1 | 0 | 1 | 0 | 0 | CD | 0 | CD |
| 8. Was the differential drop-out rate (between treatment groups) at endpoint 15 percentage points or lower? | 1 | CD | 1 | 1 | 1 | 0 | CD | 0 | 1 |
| 9. Was there high adherence to the intervention protocols for each treatment group? | 1 | 1 | 1 | 1 | 1 | 1 | 1 | 1 | 1 |
| 10. Were other interventions avoided or similar in the groups (e.g., similar background treatments)? | CD | CD | CD | CD | CD | CD | CD | CD | CD |
| 11. Were outcomes assessed using valid and reliable measures, implemented consistently across all study participants? | 1 | 1 | 1 | 1 | 1 | 1 | 1 | 1 | 1 |
| 12. Did the authors report that the sample size was sufficiently large to be able to detect a difference in the main outcome between groups with at least 80% power? | 0 | 0 | 1 | 0 | 1 | 1 | 0 | 1 | 0 |
| 13. Were outcomes reported or subgroups analyzed prespecified (i.e., identified before analyses were conducted)? | CD | CD | CD | CD | CD | CD | CD | CD | CD |
| 14. Were all randomized participants analyzed in the group to which they were originally assigned, i.e., did they use an intention-to-treat analysis? | CD | CD | CD | CD | CD | CD | CD | CD | CD |
| **Total Score** | 5 | 5 | 6 | 6 | 7 | 5 | 3 | 5 | 5 |
| **Percentile** | 29.46 | 29.46 | 49.11 | 49.11 | 67.86 | 29.46 | 4.46 | 29.46 | 29.46 |
| **Rating** | Poor | Poor | Fair | Fair | Fair | Poor | Poor | Poor | Poor |
|  |  |  |  |  |  |  |  |  |  |
|  | *Note*: CD = Cannot Determine. Ratings were based on percentile and 0-33.99% were rated as “Poor, 34-67.99% as “Fair” and 68-100% as “Good.” | | | | | | | | |

| **Criteria** | Haley et al (2024) | Harris et al (1991) | Hilbert (2016) | Huelleman et al (2023) | Joseph & Raque (2023) | Koball & Carels (2015) | Kramer et al (2024) | Kreynin et al (2024) | Ksinan et al (2017) |
| --- | --- | --- | --- | --- | --- | --- | --- | --- | --- |
| 1. Was the study described as randomized, a randomized trial, a randomized clinical trial, or an RCT? | 1 | 1 | 1 | 1 | 1 | 1 | 1 | 1 | 1 |
| 2. Was the method of randomization adequate (i.e., use of randomly generated assignment)? | 1 | CD | CD | 1 | 1 | CD | CD | CD | 1 |
| 3. Was the treatment allocation concealed (so that assignments could not be predicted)? | CD | CD | CD | CD | CD | CD | CD | CD | CD |
| 4. Were study participants and providers blinded to treatment group assignment? | CD | CD | CD | CD | CD | CD | CD | 0 | CD |
| 5. Were the people assessing the outcomes blinded to the participants' group assignments? | CD | CD | CD | CD | CD | CD | CD | CD | CD |
| 6. Were the groups similar at baseline on important characteristics that could affect outcomes (e.g., demographics, risk factors, co-morbid conditions)? | 1 | CD | 1 | CD | 0 | CD | 0 | CD | CD |
| 7. Was the overall drop-out rate from the study at endpoint 20% or lower of the number allocated to treatment? | 0 | CD | CD | 1 | 1 | CD | 1 | 1 | 1 |
| 8. Was the differential drop-out rate (between treatment groups) at endpoint 15 percentage points or lower? | 1 | CD | CD | 1 | 1 | 1 | 1 | 1 | 1 |
| 9. Was there high adherence to the intervention protocols for each treatment group? | 1 | 1 | 1 | 1 | 1 | 1 | 1 | 1 | 1 |
| 10. Were other interventions avoided or similar in the groups (e.g., similar background treatments)? | CD | CD | CD | CD | CD | CD | CD | CD | CD |
| 11. Were outcomes assessed using valid and reliable measures, implemented consistently across all study participants? | 1 | 1 | 1 | 1 | 1 | 1 | 1 | 1 | 1 |
| 12. Did the authors report that the sample size was sufficiently large to be able to detect a difference in the main outcome between groups with at least 80% power? | 0 | 0 | 1 | 1 | 0 | 0 | 1 | 0 | CD |
| 13. Were outcomes reported or subgroups analyzed prespecified (i.e., identified before analyses were conducted)? | CD | CD | CD | CD | CD | CD | CD | CD | CD |
| 14. Were all randomized participants analyzed in the group to which they were originally assigned, i.e., did they use an intention-to-treat analysis? | CD | CD | CD | 1 | CD | CD | CD | CD | CD |
| **Total Score** | 6 | 3 | 5 | 8 | 6 | 4 | 6 | 5 | 6 |
| **Percentile** | 49.11 | 4.46 | 29.46 | 83.93 | 49.11 | 13.39 | 49.11 | 29.46 | 49.11 |
| **Rating** | Fair | Poor | Poor | Good | Fair | Poor | Fair | Poor | Fair |
|  |  |  |  |  |  |  |  |  |  |
|  | *Note*: CD = Cannot Determine. Ratings were based on percentile and 0-33.99% were rated as “Poor, 34-67.99% as “Fair” and 68-100% as “Good.” | | | | | | | | |

| **Criteria** | Lee et al (2024) | Lin & Stutts (2020) | Lopez et al (2024) | Martingano et al (2023) | Matharu et al (2014) | Myre et al (2020) | Nickel et al (2019) | Nutter et al (2018) | O'Brien et al (2010) |
| --- | --- | --- | --- | --- | --- | --- | --- | --- | --- |
| 1. Was the study described as randomized, a randomized trial, a randomized clinical trial, or an RCT? | 1 | 1 | 1 | 1 | 1 | 1 | 1 | 1 | 1 |
| 2. Was the method of randomization adequate (i.e., use of randomly generated assignment)? | CD | CD | CD | CD | 1 | CD | CD | 1 | CD |
| 3. Was the treatment allocation concealed (so that assignments could not be predicted)? | CD | CD | CD | CD | CD | CD | CD | CD | CD |
| 4. Were study participants and providers blinded to treatment group assignment? | CD | CD | CD | CD | CD | CD | CD | CD | CD |
| 5. Were the people assessing the outcomes blinded to the participants' group assignments? | CD | CD | CD | CD | CD | CD | CD | CD | CD |
| 6. Were the groups similar at baseline on important characteristics that could affect outcomes (e.g., demographics, risk factors, co-morbid conditions)? | 1 | 1 | CD | CD | 1 | 1 | 0 | CD | 0 |
| 7. Was the overall drop-out rate from the study at endpoint 20% or lower of the number allocated to treatment? | 1 | 1 | 0 | 1 | 1 | 1 | CD | 0 | 1 |
| 8. Was the differential drop-out rate (between treatment groups) at endpoint 15 percentage points or lower? | 1 | 1 | 1 | CD | 1 | 1 | CD | CD | 1 |
| 9. Was there high adherence to the intervention protocols for each treatment group? | 1 | 1 | 1 | 1 | 1 | 1 | 1 | 1 | 1 |
| 10. Were other interventions avoided or similar in the groups (e.g., similar background treatments)? | CD | CD | CD | CD | CD | CD | CD | CD | CD |
| 11. Were outcomes assessed using valid and reliable measures, implemented consistently across all study participants? | 1 | 1 | 1 | 1 | 1 | 1 | 1 | 1 | 1 |
| 12. Did the authors report that the sample size was sufficiently large to be able to detect a difference in the main outcome between groups with at least 80% power? | 0 | 1 | 0 | 1 | 1 | 1 | 0 | 1 | 0 |
| 13. Were outcomes reported or subgroups analyzed prespecified (i.e., identified before analyses were conducted)? | 1 | CD | CD | 1 | CD | 0 | 0 | 1 | 0 |
| 14. Were all randomized participants analyzed in the group to which they were originally assigned, i.e., did they use an intention-to-treat analysis? | CD | CD | CD | CD | CD | 0 | 0 | 0 | CD |
| **Total Score** | 7 | 7 | 4 | 6 | 8 | 7 | 3 | 6 | 5 |
| **Percentile** | 67.86 | 67.86 | 13.39 | 49.11 | 83.93 | 67.86 | 4.46 | 49.11 | 29.46 |
| **Rating** | Fair | Fair | Poor | Fair | Good | Fair | Poor | Fair | Poor |
|  |  |  |  |  |  |  |  |  |  |
|  | *Note*: CD = Cannot Determine. Ratings were based on percentile and 0-33.99% were rated as “Poor, 34-67.99% as “Fair” and 68-100% as “Good.” | | | | | | | | |

| **Criteria** | O'Brien et al (2020) | Oliver et al (2022) | Palmeria et al (2017) | Pearl et al (2020) | Pearl et al. (2023) | Persky et al (2011) | Potts et al (2022) | Rodriguez et al (2016) | Rosenbaum (2024) |
| --- | --- | --- | --- | --- | --- | --- | --- | --- | --- |
| 1. Was the study described as randomized, a randomized trial, a randomized clinical trial, or an RCT? | 1 | 1 | 1 | 1 | 1 | 1 | 1 | 1 | 1 |
| 2. Was the method of randomization adequate (i.e., use of randomly generated assignment)? |  | CD | 1 | CD | 1 | CD | 1 | 1 | 1 |
| 3. Was the treatment allocation concealed (so that assignments could not be predicted)? | CD | CD | CD | CD | CD | CD | CD | CD | CD |
| 4. Were study participants and providers blinded to treatment group assignment? | CD | 1 | 0 | 0 | 0 | CD | CD | 0 | CD |
| 5. Were the people assessing the outcomes blinded to the participants' group assignments? | CD | CD | CD | 0 | 0 | CD | CD | 0 | CD |
| 6. Were the groups similar at baseline on important characteristics that could affect outcomes (e.g., demographics, risk factors, co-morbid conditions)? | CD | 1 | 0 | 1 | 1 | 1 | 1 | 1 | 1 |
| 7. Was the overall drop-out rate from the study at endpoint 20% or lower of the number allocated to treatment? | 1 | 1 | 1 | 1 | 1 | CD | 0 | 1 | 1 |
| 8. Was the differential drop-out rate (between treatment groups) at endpoint 15 percentage points or lower? | CD | 1 | 1 | 1 | 1 | CD | 1 | 1 | 1 |
| 9. Was there high adherence to the intervention protocols for each treatment group? | 1 | 1 | 1 | 1 | 1 | 1 | 1 | 1 | 1 |
| 10. Were other interventions avoided or similar in the groups (e.g., similar background treatments)? | CD | CD | CD | CD | CD | CD | CD | CD | CD |
| 11. Were outcomes assessed using valid and reliable measures, implemented consistently across all study participants? | 1 | 1 | 1 | 1 | 1 | 1 | 1 | 1 | 1 |
| 12. Did the authors report that the sample size was sufficiently large to be able to detect a difference in the main outcome between groups with at least 80% power? | 0 | 0 | 1 | 1 | 1 | 0 | 0 | 1 | 0 |
| 13. Were outcomes reported or subgroups analyzed prespecified (i.e., identified before analyses were conducted)? | CD | 0 | 1 | 1 | 1 | CD | CD | 1 | CD |
| 14. Were all randomized participants analyzed in the group to which they were originally assigned, i.e., did they use an intention-to-treat analysis? | CD | CD | 1 | 1 | 1 | CD | 1 | CD | CD |
| **Total Score** | 4 | 7 | 9 | 9 | 10 | 4 | 7 | 9 | 7 |
| **Percentile** | 13.39 | 67.86 | 92.86 | 92.86 | 98.21 | 13.39 | 67.86 | 92.86 | 67.86 |
| **Rating** | Poor | Fair | Good | Good | Good | Poor | Fair | Good | Fair |
|  |  |  |  |  |  |  |  |  |  |
|  | *Note*: CD = Cannot Determine. Ratings were based on percentile and 0-33.99% were rated as “Poor, 34-67.99% as “Fair” and 68-100% as “Good.” | | | | | | | | |

| **Criteria** | Rudolph & Hilbert (2017) | Sherf-Dagan et al (2022) | Sherf-Dagan et al (2024) | Speirs et al (2022) | Swift et al (2013) | Turner et al (2012) | Turner et al (2022) | Wiese et al (1992) | Wijayatunga et al (2021) |
| --- | --- | --- | --- | --- | --- | --- | --- | --- | --- |
| 1. Was the study described as randomized, a randomized trial, a randomized clinical trial, or an RCT? | 1 | 1 | 1 | 1 | 1 | 1 | 1 | 1 | 1 |
| 2. Was the method of randomization adequate (i.e., use of randomly generated assignment)? | CD | 1 | 1 | 1 | 1 | CD | CD | 1 | 1 |
| 3. Was the treatment allocation concealed (so that assignments could not be predicted)? | CD | CD | CD | CD | CD | CD | CD | CD | CD |
| 4. Were study participants and providers blinded to treatment group assignment? | CD | CD | CD | CD | CD | CD | CD | CD | CD |
| 5. Were the people assessing the outcomes blinded to the participants' group assignments? | CD | CD | CD | CD | CD | CD | CD | CD | 0 |
| 6. Were the groups similar at baseline on important characteristics that could affect outcomes (e.g., demographics, risk factors, co-morbid conditions)? | CD | 1 | 1 | CD | 1 | CD | CD | 1 | 1 |
| 7. Was the overall drop-out rate from the study at endpoint 20% or lower of the number allocated to treatment? | CD | 0 | 1 | CD | 0 | CD | CD | CD | 0 |
| 8. Was the differential drop-out rate (between treatment groups) at endpoint 15 percentage points or lower? | CD | 1 | 1 | CD | 1 | CD | CD | CD | 1 |
| 9. Was there high adherence to the intervention protocols for each treatment group? | 1 | 1 | 1 | 1 | 1 | 1 | 1 | 1 | 1 |
| 10. Were other interventions avoided or similar in the groups (e.g., similar background treatments)? | CD | CD | CD | CD | CD | CD | CD | CD | CD |
| 11. Were outcomes assessed using valid and reliable measures, implemented consistently across all study participants? | 1 | 1 | 1 | 1 | 1 | 1 | 1 | 1 | 1 |
| 12. Did the authors report that the sample size was sufficiently large to be able to detect a difference in the main outcome between groups with at least 80% power? | 0 | 0 | 1 | 1 | 0 | 0 | 1 | 0 | 1 |
| 13. Were outcomes reported or subgroups analyzed prespecified (i.e., identified before analyses were conducted)? | CD | 1 | 1 | CD | CD | CD | CD | CD | 1 |
| 14. Were all randomized participants analyzed in the group to which they were originally assigned, i.e., did they use an intention-to-treat analysis? | CD | 1 | 1 | CD | 1 | CD | CD | CD | CD |
| **Total Score** | 3 | 8 | 10 | 5 | 7 | 3 | 4 | 5 | 8 |
| **Percentile** | 4.46 | 83.93 | 98.21 | 29.46 | 67.86 | 4.46 | 13.39 | 29.46 | 83.93 |
| **Rating** | Poor | Good | Good | Poor | Fair | Poor | Poor | Poor | Good |
|  |  |  |  |  |  |  |  |  |  |
|  | *Note*: CD = Cannot Determine. Ratings were based on percentile and 0-33.99% were rated as “Poor, 34-67.99% as “Fair” and 68-100% as “Good.” | | | | | | | | |

| **Criteria** | Wilson et al (2020) | Zuest et al (2024) |  |  |  |  |  |  |  |
| --- | --- | --- | --- | --- | --- | --- | --- | --- | --- |
| 1. Was the study described as randomized, a randomized trial, a randomized clinical trial, or an RCT? | 1 | 1 |  |  |  |  |  |  |  |
| 2. Was the method of randomization adequate (i.e., use of randomly generated assignment)? | CD | 1 |  |  |  |  |  |  |  |
| 3. Was the treatment allocation concealed (so that assignments could not be predicted)? | CD | CD |  |  |  |  |  |  |  |
| 4. Were study participants and providers blinded to treatment group assignment? | CD | CD |  |  |  |  |  |  |  |
| 5. Were the people assessing the outcomes blinded to the participants' group assignments? | CD | CD |  |  |  |  |  |  |  |
| 6. Were the groups similar at baseline on important characteristics that could affect outcomes (e.g., demographics, risk factors, co-morbid conditions)? | 1 | CD |  |  |  |  |  |  |  |
| 7. Was the overall drop-out rate from the study at endpoint 20% or lower of the number allocated to treatment? | 0 | 0 |  |  |  |  |  |  |  |
| 8. Was the differential drop-out rate (between treatment groups) at endpoint 15 percentage points or lower? | 0 | 1 |  |  |  |  |  |  |  |
| 9. Was there high adherence to the intervention protocols for each treatment group? | 1 | 1 |  |  |  |  |  |  |  |
| 10. Were other interventions avoided or similar in the groups (e.g., similar background treatments)? | CD | CD |  |  |  |  |  |  |  |
| 11. Were outcomes assessed using valid and reliable measures, implemented consistently across all study participants? | 1 | 1 |  |  |  |  |  |  |  |
| 12. Did the authors report that the sample size was sufficiently large to be able to detect a difference in the main outcome between groups with at least 80% power? | 1 | 0 |  |  |  |  |  |  |  |
| 13. Were outcomes reported or subgroups analyzed prespecified (i.e., identified before analyses were conducted)? | CD | CD |  |  |  |  |  |  |  |
| 14. Were all randomized participants analyzed in the group to which they were originally assigned, i.e., did they use an intention-to-treat analysis? | CD | CD |  |  |  |  |  |  |  |
| **Total Score** | 5 | 5 |  |  |  |  |  |  |  |
| **Percentile** | 29.46 | 29.46 |  |  |  |  |  |  |  |
| **Rating** | Poor | Poor |  |  |  |  |  |  |  |
|  |  |  |  |  |  |  |  |  |  |
|  | *Note*: CD = Cannot Determine. Ratings were based on percentile and 0-33.99% were rated as “Poor, 34-67.99% as “Fair” and 68-100% as “Good.” | | | | | | | | |
